# Deciphering the *Streptococcus mutans* essentialome: multi-omic resolution of hypothetical genes and identification of a functional RocS equivalent

**DOI:** 10.64898/2026.07.15.738700

**Published:** 2026-07-16

**Authors:** Courtney E. Dover, Kipa Tamrakar, Bikash Dwivedi, Evan R. Roberts, Sangam Chudal, Shawn King, Emilio Soriano Chavez, Valérie de Crécy-Lagard, Robert C. Shields

## Abstract

Genome-wide viability catalogs produced by transposon sequencing (Tn-seq) and CRISPR interference (CRISPRi) have successfully mapped the essential genome of *Streptococcus mutans* . However, particularly for genes annotated as “hypothetical” or uncharacterized, translating these findings into mechanistic biological functions remains a significant bottleneck. In this study, we developed an integrated functional genomics pipeline combining predictive bioinformatics, tunable CRISPRi transcriptional silencing, transmission electron microscopy, transcriptomics, and genetic suppressor screens to characterize nine legacy hypothetical essential genes in *S. mutans* . Comparative transcriptomics and proteomics revealed a conserved baseline stress signature across diverse essential pathways, marked by the coordinated downregulation of the *citZ* - *citB* - *idh* metabolic locus and insoluble matrix synthesis enzymes ( *gtfBC* ), paired with the robust activation of the integrative and conjugative element Tn *Smu1* . Against this backdrop of systemic stress, we successfully resolved the function of SMU_393, defining it as a functional equivalent of the pneumococcal regulator of chromosome segregation, RocS. Depletion of SMU_393 resulted in abnormal cell widening, hypersensitivity to DNA damage, and a significant subpopulation of anucleate cells. Remarkably, these phenotypes were bypassed by a spontaneous surface-exposed missense mutation ( *dnaA* ^Q197E^ ) within the AAA+ ATPase domain of the replication initiator. Together, this work uncovers an important cell cycle regulator and provides a framework for exploring uncharacterized essential genes of the oral microbiome.

**Importance:**

Although genome sequencing has identified thousands of genes required for bacterial survival, the precise biological roles for many of them remain completely unknown. This study implements an integrated functional genomics pipeline to resolve the molecular functions of legacy uncharacterized essential genes in the oral pathogen *Streptococcus mutans* . We discovered a critical molecular checkpoint that acts as a physical anchor, linking the bacterial chromosome to the cell envelope to ensure that chromosome replication is synchronized with cell division. Remarkably, a single mutation in the replication machinery can fully bypass the loss of this anchor, maintaining proper genetic inheritance even during severe cellular stress. Ultimately, this study provides a pipeline for uncovering highly specific physiological vulnerabilities that can be exploited for targeted therapeutics against oral pathogens.

## Full Text Availability

The license terms selected by the author(s) for this preprint version do not permit archiving in PMC. The full text is available from the preprint server.

